# Direct hospital costs of chest pain patients attending the emergency department: a retrospective study

**DOI:** 10.1186/1471-227X-6-6

**Published:** 2006-05-04

**Authors:** Jakob L Forberg, Louise S Henriksen, Lars Edenbrandt, Ulf Ekelund

**Affiliations:** 1 Department of Emergency Medicine, Lund University Hospital, Lund, Sweden; 2 Copenhagen Business School, Copenhagen, Denmark; 3 Department of Clinical Physiology, Malmö University Hospital, Malmö, Sweden; 4 Department of Clinical Physiology, Sahlgrenska University Hospital, Gothenburg, Sweden

## Abstract

**Background:**

Chest pain is one of the most common complaints in the Emergency Department (ED), but the cost of ED chest pain patients is unclear. The aim of this study was to describe the direct hospital costs for unselected chest pain patients attending the emergency department (ED).

**Methods:**

1,000 consecutive ED visits of patients with chest pain were retrospectively included. Costs directly following the ED visit were retrieved from the hospital economy system.

**Results:**

The mean cost per patient visit was 26.8 thousand Swedish kronar (kSEK) (median 7.2 kSEK), with admission time accounting for 73% of all costs. Mean cost for patients discharged from the ED was 1.4 kSEK (median 1.3 kSEK), and for patients without ACS admitted 1 day or less 7.6 kSEK (median 6.9 kSEK). The practice in the present study to admit 67% of the patients, of whom only 31% proved to have ACS, was estimated to give a cost per additional life-year saved by hospital admission, compared to theoretical strategy of discharging all patients home, of about 350 kSEK (39 kEUR or 42 kUSD).

**Conclusion:**

Costs for chest pain patients are large and primarily due to admission time. The present admission practice seems to be cost-effective, but the substantial overadmission indicates that better ED diagnostics and triage could decrease costs considerably.

## Background

Patients attending the emergency department (ED) with chest pain indicating a possible acute coronary syndrome (ACS) are very common. In Sweden (population 9 million), some 180,000 patients with suspected ACS present to EDs every year [1]. Treatment of ACS has improved dramatically over the last two decades, but it has also become time-dependent, and the prognosis of the patient now depends on accurate management decisions made already at the ED. In addition, the therapy for ACS has become costly, with new drugs and balloon angioplasty using advanced stents.

Because of the costly therapy and the large size of the patient category, correct management decisions in the ED are probably also of great economic importance. Though there are numerous studies describing costs for subgroups of chest pain patients [2-10], there are few data regarding the economic consequences of ED decisions in an unselected chest pain patient population [11]. With such data available, it seems reasonable that hospital resources could be better allocated to and within this large patient group, and that more rational development of chest pain management programs would be possible.

The aim of this study was to describe the direct hospital costs for unselected chest pain patients who attend the ED, focusing on the economic consequences of the decisions by the ED physician to hospitalize the patient, and to refer the patient to different diagnostic procedures and levels of in-hospital care. An additional aim was to estimate the cost per additional life-year saved by hospital admission, compared to theoretical strategy of discharging all patients home.

## Methods

### Subjects

The study retrospectively included 1000 consecutive patients with chest pain attending the ED at Lund University Hospital, Sweden, from July 1 to November 20, 1997. Lund University Hospital is a 1200 bed institution with fully public financing that serves a population of some 250,000. The ED has about 47000 visits a year. Percutaneous coronary intervention (PCI) and coronary bypass surgery (CABG) are available 24 hours/day. During the patient inclusion period, there was no systematic diagnostic protocol for patients with suspected ACS, no dedicated chest pain unit, and no formal strategy for admitting ED patients to in-hospital care.

### Data collection

Using the computerized ED information system, consecutive patients with a presenting complaint of chest pain or discomfort (e.g. tightness, pressure, "angina") as noted by the initial triage nurse, were retrospectively included in the study. Patient data were collected from the medical records by an experienced research nurse. The discharge diagnoses were made by the attending senior ward physicians, and also reviewed by a senior research nurse. A patient was considered having ACS when discharged with a diagnosis of either AMI or unstable angina. AMI was defined by the WHO criteria [12] where the biochemical criterion was at least one measurement of CK-MB>10 μg/l or Troponin T>0,1 μg/l. The criteria for unstable angina were: 1) Ischemic symptoms (chest pain >15 min., syncope, acute heart failure or pulmonary oedema) with one of the following: 2) electrocardiogram (ECG) changes: transient or persisting ST segment depression (≥ 1 mm) and/or T-wave inversion (≥ 1 mm) without developing Q waves or loss of R wave height, 3) Biochemical markers CK-MB 5 – 10 *μ*g/l or Troponin T 0.05 – 0.1 *μ*g/l.

Only costs following directly from each ED visit until hospital discharge were included. All costs were taken from the hospital's computerized economy system METIS. All unit costs were set by the Lund University Hospital economy department and were the yearly calculated average unit costs. The unit cost for the ED visit and the cost per admission day at the respective wards include overheads, capital costs, staff costs and pharmaceutical costs. The total cost for each included patient was calculated as the sum of the cost of the ED and hospital admission (when applicable) and the costs of performed procedures and diagnostic tests (laboratory tests, x-ray, exercise test etc.). All costs are from 1997 (no up-rating for inflation) and are presented in kSEK. In December 2005, 1 SEK equalled 0.11 EUR and 0.12 USD.

### Assumptions

In order to evaluate the cost-effectiveness of our current practice, we made an attempt to estimate the cost of additional life-years saved with the current admission practice compared to a theoretical strategy of discharging all patients from the ED. Using results from the Multicenter Chest pain study [13] the excess mortality after an ACS were estimated to be 4.9%, 3.6%, 1.6% for patients 1, 2 and 3 or more years after discharge, respectively [2]. This increased yearly mortality was applied to a Swedish life table from 1999 [14] and the life-years lost for each patients with ACS who died in the present material were calculated. Previous studies suggest that failure to admit patients with myocardial infarction [15,16] or unstable angina [16], increases short term mortality about two-fold. Thus mortality from ACS was assumed to have approximately doubled with the theoretical discharge-all strategy, as has been assumed in previous studies [3,17]. The gain from the actual admission practice compared to a discharge-all strategy, i.e. additional life-years saved, was thus estimated to equal the calculated life-years lost with the actual admission practice. It should be noted, however, that this assumption does not include potential benefits from PCI therapy to patients with ST-elevation AMI.

### Ethical approval and statistics

The Research Ethics Committee at Lund University approved the study. Data was analysed using the SPSS 12.01 statistical software package. Data are presented as mean and median. For statistical comparison of means, the t-test was used when comparing two groups and the ANOVA test were used when comparing multiple groups. Differences were considered significant at P < 0.05.

## Results

### Patient characteristics and overall cost

Selected patient characteristics are presented in Table 1. 813 patients had only one visit and 68 patients had two visits or more.

**Table 1:** Characteristics of the chest pain patients included in the present study with a total of 1,000 ED visits. CABG, coronary artery bypass grafting; PCI, percutaneous coronary intervention

	**All patients **(n = 881)
**Median age **(percentile 05–95)	65 (31–87)
**Male**	57%
**Smoking**	22%
**Hypertension**	31%
**Diabetes**	11%
**Congestive heart failure**	15%
**Previous angina pectoris**	38%
**Previous myocardial infarction**	28%
**Previous PCI**	6%
**Previous CABG**	10%

The total cost for all patient visits was 26.8 million (M) SEK. The mean cost per visit was thus 26.8 kSEK (k2.9 EUR or k3.2 USD), with a median cost of 7.2 kSEK. Fifty-eight percent of the cases cost less than 10 kSEK, and only 1.3% of the patient visits cost more than 250 kSEK. In Table 2 is presented a selection of used in-hospital resources, and in Table 3 is a selection of unit costs presented.

**Table 2:** Examples of in-hospital resource use.

	**Number per 1000 ED visits**
**Admitted to CCU**	190
**Admitted to ICW**	408
**Admitted to GW**	63
**CABG**	15
**PCI**	27
**Coronary angiography**	48
**Thrombolysis**	41
**Exercise test**	90
**Echocardiography**	153

CCU, Cardiac care unit; ICW, Intermediate care ward; GW, general ward; CABG, coronary artery bypass grafting; PCI, percutaneous coronary intervention

**Table 3:** Examples of unit costs in kSEK, and in corresponding EUR.

	**Costs**	
	**kSEK**	**kEUR**

**Visit at the ED**	0.9	0.1
**CCU (per day)**	10.6	1.2
**ICW (per day)**	4.6	0.5
**GW (per day)**	2.6	0.3
**CABG**	103	11.3
**PCI with/without stent**	35.3/24.2	3.9/2.7
**Coronary angiography**	3	0.3
**Exercise test**	1.1	0.1
**Echocardiography**	1.1	0.1

ED, Emergency department; CCU, Cardiac care unit; ICW, Intermediate care ward; GW, general ward; CABG, coronary artery bypass grafting; PCI, percutaneous coronary intervention

### The decision to discharge or admit

**Figure 1:**
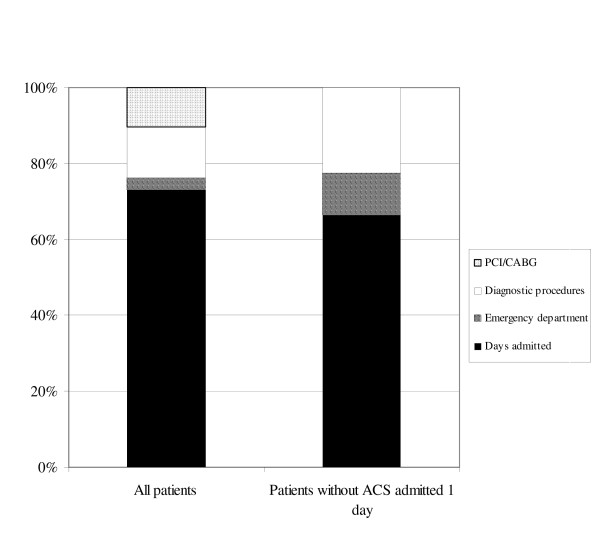
Comparison of the composition of total costs for all patients (left bar), and patients without ACS admitted for one day or less (right bar).

One of the primary decisions to be made by the ED physician is whether to admit the patient to in-hospital care or not. A total of 661 patients were hospitalized, and the length of stay varied from 1 to 143 days with a mean of 5.3 days (median 2 days). As can be seen in Figure 1, left bar, the decision to admit is usually an economically important one; Admission days accounted for 73% and diagnostic procedures (different tests and x-ray) for only 13% of the total costs.

In chest pain patients perceived to have a low risk of ACS, the ED physician has the option either to immediately discharge the patient from the ED or to admit the patient for an in-hospital "ACS rule-out" observation. The latter was common in the present study, since an ACS was diagnosed in only 207 (31%) of the admitted patients. Four hundred fifty-four patients without a final ACS diagnosis were admitted, and 244 of these patients (54%) were discharged after one day or less. The distribution of costs for these patients are shown in Figure 1, right bar. The mean total cost was 7.6 kSEK (median 6.9 kSEK), with admission time accounting for 66% and diagnostic procedures (different tests and x-ray) for 22% of these costs. Patients discharged from the ED cost a mean 1.4 kSEK (median 1.3 kSEK). For the patients without ACS, the difference between the mean costs of a "rule-out" observation and immediate discharge was thus 6.2 kSEK.

### Costs in relation to level of care

**Figure 2:**
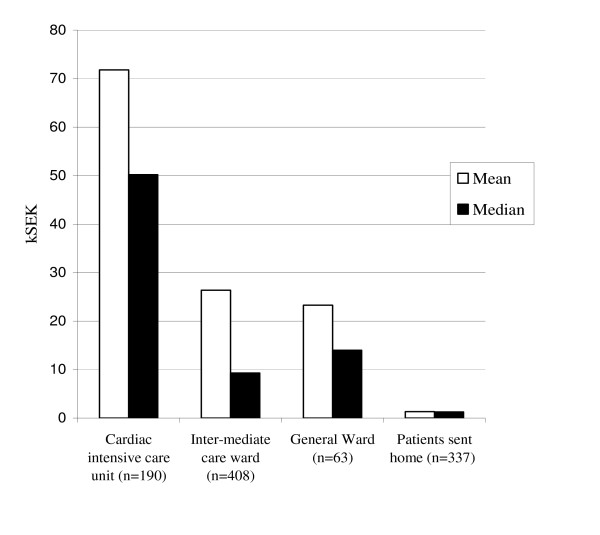
Total cost per visit in relation to ED triage to discharge (right bars) and admission to different hospital units. N = number of patient visits in each group. Two patients that died in the ED are not included.

When the decision has been made to admit the patient, the next question is bed assignment. Total costs for patients assigned to different levels of care are presented in Figure 2. The proportions of patients discharged with a diagnosis of ACS were for the Coronary Care Unit (CCU), Intermediate Care Ward (ICW) and General Ward (GW) 60%, 23% and 5%, respectively. Costs for diagnostic procedures were significantly higher in the CCU (mean 7.8 kSEK, median 3.4 kSEK) than in the ICW (mean 3.6 kSEK) or GW (mean 4.1 kSEK).

### Costs in relation to discharge diagnosis

Patients with ACS (n = 207) as expected generated significantly higher costs (mean 79.9, median 56.5 kSEK) than those without ACS (n = 791; mean 12.8, median 6.4 kSEK). Patients with ACS accounted for 62% of the total cost for all the included patients. Patients with AMI cost a mean 84,4 kSEK (median 59,2 kSEK).

### Cost per life-year saved

The in-hospital mortality for patients with ACS was 5.3% (11 patients of which two died in the ED) and the total life-years lost were estimated to be 77 years. Assuming a two-fold increase in mortality and life-years lost if no patients would have been admitted, the cost of one additional life-year saved with the current hospitalization practice, compared to dismissing all patients from the ED, was estimated to be some 350 kSEK (39 kEUR or 42 kUSD)

## Discussion

Since chest pain is one of the most common complaints in the ED, the cost of these patients has a significant impact on total health care costs. The present study indicates that costs for chest pain patients are large and primarily due to admission time. Extrapolation of the present average per-visit cost of 26.8 kSEK yields a cost of 275 MSEK per million inhabitants and year for the evaluation of patients presenting at the ED with chest pain. Since there has been a general cost increase about 30% at Lund University Hospital from 1997 to 2005, we estimate the current cost to be approximately 360 MSEK per million inhabitants. In addition to the general cost increase at our hospital, a number of changes in chest pain management since 1997 have likely affected cost development. First, there may have been an increase in hospitalization of low risk chest pain patients since 1997 [18], which can contribute to a total cost increase. Second and more importantly, there has been an increased use of coronary angiography and PCI, and a decreased use of thrombolysis [19]. The Swedish national register for coronary care units (RIKS-HIA) shows an unchanged rate of reperfusion therapy for ST-elevation myocardial infarction (STEMI) patients from 1997 to 2003, but a overall nation-wide increase in the use of primary PCI from about 5% to 20 %[20]. In 2003, Swedish academic medical centres with revascularization facilities (like our institution) performed primary PCI in more than 50% of STEMI patients [20]. For non-STEMI patients, inpatient coronary angiography increased from 25% to 62% during 1997 – 2003. This increased proportion of costly invasive procedures has thus probably increased the total hospital cost of chest pain patients since 1997. However, the more frequent use of invasive procedures applies almost only to the minority of patients that have ACS (21% in the present material), whereas the management of the majority of the patients (without ACS) is almost unchanged, at least in Sweden. It is therefore reasonable to believe that patients with ACS now contribute with more than 62 % of the total cost. The patterns of cost presented here should thus be applicable to the majority of the patients in 2006, and could be used for e.g. economic evaluations of new diagnostic methods for low/moderate risk chest pain patients in the ED.

### Comparison with previous studies

Few previous studies describe the cost of consecutive, unselected ED patients [10,11]. The study most similar to the present one is by Kontos et al [11] 1994, which reports the 30 days cost of consecutive ED patients with symptoms suggestive of acute cardiac ischemia. In this study, patients managed with a standard diagnostic strategy cost an average $6044 (43 kSEK), which is 60 % higher than in the present study, despite a shorter length of stay (3 vs 5 days) and a lower proportion of MI's (10 vs 13%). On the other hand, the cost of patients without ACS admitted one day or less (7.6 kSEK) in the present study is comparable to the cost of non-chest pain unit care (7.1 kSEK) for patients without ACS in a UK study [4]. The cost for chest pain unit (CPU) care for similar patients in the US has been higher; $1–$9 k, or 7–64 kSEK [7-9,21]. Further, our ACS patients (79.9 kSEK) cost somewhat more than AMI patients in Europe (51–70 kSEK [22,23]), but somewhat less than low risk AMI patients in the USA ($10–13.5 k, or 86–95 kSEK [6,7]). Possible explanations for these differences include different cost sampling periods, reimbursement systems, triage routines and resource utilization patterns.

### Cost effectiveness

Our practice to admit 67% of the ED chest pain patients, with 31 % of the admitted having an ACS, was crudely estimated to result in a cost of about 350 kSEK (equalling approximately 38 kEUR) per life-year saved compared to a theoretical strategy of discharging all patients home from the ED. According to the Swedish National Board of Health and Welfare, this is regarded a cost-effective practice (< 500 kSEK per life-year saved). Our results are thus consistent with the simulation model of Wears et al. which indicated that an AMI rate of 20–30% or more among the admitted patients is cost-effective[5]. In the present study, the number of additional life-years saved was crudely estimated from the in-hospital reduction in mortality only in ACS patients, and not in patients with other diagnoses.

Our assumption that mortality would be twice as high if ACS patients were discharged from the ED is based on two US studies from 1977–85[15] and 1993[16] which report the 3 day and 30 day mortality, respectively. The patients included in the study by Pope et al[16], were slightly different from our patients; they were on average 3–4 years younger and more had diabetes (21%), and this of course influences the validity of our assumption and the accuracy of our estimation of the cost of life-years saved. Further, the 95 percent confidence interval of the 2-fold mortality increase reported by Pope et al. was broad, 0.7 to 5.2. In the study by Lee et al, comparable patient characteristics and a confidence interval are not given. As the above studies only looked at short term mortality and were performed before modern reperfusion therapy such as PCI and secondary prophylactics, the true cost of a life-year saved by hospitalization in the present study may well be lower than our estimation.

Our current practice thus seems to be cost-effective overall, but the large overadmission indicates that there is still a great potential for cost savings and improved quality of care with improved diagnostic strategies in the ED. Also, it is likely that there are subgroups for which our admission routines would not be considered economically effective. For instance, for chest pain patients presenting with a normal ECG, the cost for a life-year saved has been estimated to be $ 1.7 million [6].

### Implications for improvement of patient management

From Figure 1 it is clear that efforts to decrease costs for suspected ACS patients should primarily be aimed at reducing the length of hospital stay. This is well in line with the current movement towards dedicated CPUs, with rapid rule-out diagnostic protocols. Randomized controlled trials on the value of these CPUs are few but indicate that they can introduce more effective patient evaluation with outcomes similar to that in traditional EDs. Cost savings have been reported per evaluated patient [4], but not yet at the hospital level.

It seems that the ideal diagnostic strategy, however, is one performed immediately in the ED, without the need for CPU or hospital admission. In the future, it may well be that modern conventional ED management supplemented by risk prediction algorithms, blood samples for new cardiac markers and/or selected investigations such as myocardial perfusion imaging [11], computerized tomography or magnetic resonance imaging will decrease or even eliminate the benefits of establishing dedicated chest pain units.

### Potential cost reductions with new ED evaluation strategies

Physicians often have multiple reasons for admitting the patient, and it has been estimated that even with perfect diagnostics in the ED, only some 40% of the now admitted patients without ACS could be immediately discharged from the ED [1]. In the present study 37% (n = 244) of the admitted patients without ACS were hospitalized for one day or less. We assume that this is the group of patients that could, with improved diagnostics in the future, be directly discharged from the ED. For each such patient discharged, some 6 kSEK could be saved (Results), if extra costs from improved diagnostics are small.

Improved decision-making in the ED could not only reduce the number of patients admitted without ACS but also reduce the number of patients admitted to a higher level of care than needed. Since 40% of patients admitted to the CCU and 77% of the patients admitted to the ICW did not have ACS, there seems to be a potential for more appropriate utilization of these units and large cost savings. Admitting a patient to the ICW instead of the CCU, or to the GW instead of the ICW would crudely halve the cost per admission day. Several studies have shown that the fraction of low and moderate risk patients admitted to non-intensive care units can safely be increased with modern risk prediction algorithms [24].

### Study limitations

Our data were retrospective and collected from July to November at only one ED in Sweden, which limit the general applicability of our results. For instance, seasonal variation in patient flow may exist. Only direct hospital costs were analyzed, which prevents any conclusion regarding the total health care or society costs for these patients. The cost of pharmaceuticals was included in the cost of admission days as a fixed percentage. The true cost of pharmaceuticals in different subgroups of patients can and does vary. The results of the cost-effectiveness analysis did not take into account the potential uncertainty in the parameters used, due to sampling variability and the assumptions used.

## Conclusion

The present results indicate that chest pain patients contribute with a high direct cost to our health care system. Our current admission practice seems to be overall cost effective. There is however still a large potential for cost savings. Almost 40% of the total cost was spent on patients not having an ACS, with admission time accounting for about 2/3 of the total cost for these patients. Efforts to reduce cost should be aimed at minimizing admissions for patients without ACS via improved ED diagnostics, and/or at reducing length of stay. Methods to safely reduce of the number of non-ACS patients admitted to the CCU could add further cost savings.

## Competing interests

The author(s) declare that they have no competing interests.

## Authors' contributions

JLF participated in the design of the study, data acquisition, data analysis, and wrote the manuscript. LSH collected and analysed data and drafted the manuscript. LE participated in the design of the study and critical revision of the manuscript. UE participated in the conception and design of the study, managed the project and wrote the manuscript. All authors have read and approved the final version of the manuscript

## Pre-publication history

The pre-publication history for this paper can be accessed here:


